# Assessment of Noise and Associated Health Impacts at Selected Secondary Schools in Ibadan, Nigeria

**DOI:** 10.1155/2009/739502

**Published:** 2009-10-08

**Authors:** Godson R. E. E. Ana, Derek G. Shendell, G. E. Brown, M. K. C. Sridhar

**Affiliations:** ^1^College of Medicine, University of Ibadan, Ibadan, Nigeria; ^2^Institute of Public Health, Georgia State University, Atlanta, GA 30302-3995, USA; ^3^Department of Environmental and Occupational Health, School of Public Health, University of Medicine and Dentistry of New Jersey (UMDNJ), Piscataway, NJ 08854, USA; ^4^Environmental and Occupational Health Sciences Institute, UMDNJ-Robert Wood Johnson Medical School, Rutgers University, Piscataway, NJ 08854, USA

## Abstract

*Background*. Most schools in Ibadan, Nigeria, are located near major roads (mobile line sources). We conducted an initial assessment of noise levels and adverse noise-related health and learning effects. *Methods*. For this descriptive, cross-sectional study, four schools were selected randomly from eight participating in overall project. We administered 200 questionnaires, 50 per school, assessing health and learning-related outcomes. Noise levels (A-weighted decibels, dBA) were measured with calibrated sound level meters. Traffic density was assessed for school with the highest measured dBA. Observational checklists assessed noise control parameters and building physical attributes. *Results*. Short-term, cross-sectional school-day noise levels ranged 68.3–84.7 dBA. Over 60% of respondents reported that vehicular traffic was major source of noise, and over 70% complained being disturbed by noise. Three schools reported tiredness, and one school lack of concentration, as the most prevalent noise-related health problems. *Conclusion*. Secondary school occupants in Ibadan, Nigeria were potentially affected by exposure to noise from mobile line sources.

## 1. Introduction

Recently, noise pollution has been of increasing concern worldwide, particularly in most urban centers. The noise problems of the modern industrial societies seem incomparable to the past given the larger sources of noise now present outdoors and indoors. According to the World Health Organization [1], traffic noise is one of the main sources of environmental noise exposure in urban communities.

Like the home and the work place, school is also an important microenvironment. The school is important for the cognitive, creative, and social development of children. Schools are therefore expected to ensure the best possible conditions for a child's physical and intellectual development, including control of excess environmental noise.

Noise levels are measured in decibels (dB). One decibel is the threshold of hearing. Approximately 60 dB is the level of normal talking. According to WHO [2], the permissible noise level in school environments should not exceed 35 dB. Exposure for more than six hours a day to sound in excess of 85 dB is potentially hazardous to health [1]. In less developed countries (LDCs) like Nigeria, many children do not have access to ideal or serene learning environments. Noise control in the school environment is a real public health challenge.

Measurements in A-weighted dB (dBA) assess loudness and compensate for the human ear's lower sensitivity to lower frequency and very high-frequency sounds [3]. Noise has both auditory and nonauditory effects [1–4]. Although the direct physical consequence of loud noise, especially over a period of time, is hearing loss and tinnitus (auditory effect), noise at lower levels can have an indirect impact on our physiological and psychological systems, that is, non auditory effects. Scientific evidence has suggested that chronic noise exposure in communities near air, road, and/or rail traffic, as a stress and distracting stimulus, can lead—among adult and children at schools and homes—to adverse health outcomes like elevated blood pressure (hypertension), noise-induced hearing loss, annoyance, stress, mental health and behavior problems, and decrease school performance and cognitive delays like trouble with word discrimination, reading, problem solving, memorization, and interference with speech communication [4, 5]. We note, however, to date that this research was primarily conducted in industrialized countries of Europe and North America, not LDCs, where most urbanization continues to occur. These health effects, in turn, can lead to social handicap, reduced productivity, decreased performance in learning, absenteeism in the workplace and school, increased drug use, and accidents [4, 5]. Furthermore, stress and hypertension are commonly regarded as being among the leading causes of population health problems. In addition, tinnitus can lead to forgetfulness, severe depression, and at times panic attacks [6]. Noise, therefore, is a physical exposure agent and environmental and occupational hazard presenting risks to our overall health and well-being.

 Studies carried out in industrialized country cities such as in the European Union have suggested that children living and attending schools near airports, elevated trains, and highways suffer distractions, lack of concentration, and restlessness, resulting in poor scores and lower productivity in their academic performances as compared to their peers in less noisy environments [7–14]. In LDCs where urban laws and proper land use conditions that either do not exist or are not always monitored and enforced, few locale-specific data exist to help improve the situation.

The objective of this pilot study was to initially assess cross-sectional indoor and outdoor environmental noise levels and noise-related health and learning outcomes including symptoms at urban schools in an LDC in Africa. We focused on selected secondary schools in the city of Ibadan, an indigenous highly populated and still developing and sprawling urban area in southwestern Nigeria, West Africa. Most schools are near major roadways with automobiles, buses, and large trucks and oil tankers.

## 2. Materials and Methods

This study went through proper required institutional review board procedures at the College of Medicine, University of Ibadan prior to its initiation. Informed consent was obtained from participating study schools.

We conducted the overall study in eight secondary schools; this paper focused on selected noise and health and learning related data from four study schools. We received permission to refer to schools by acronyms as defined below.

### 2.1. Study Area

Ibadan is the capital of Oyo State in Nigeria and one of the largest cities (metropolitan areas) in West Africa, with millions of inhabitants. Ibadan is an old, primarily indigenous African city situated between latitude 7° and 9°30′ east of prime meridian. Ibadan covers a large land area of about 12 square kilometers at an altitude ranging from about 150 to over 200 meters with isolated ridges and peaks rising over about 270 meters. Ibadan presents a typical picture of many African cities each known for having the old town area (inner core) and then the transitional and peripheral areas. Most people are Yoruba; other ethnic groups constitute smaller proportions of the population. Ibadan has over 300 academic settings comprised of public and private nursery, primary schools and secondary schools as well as a university.

### 2.2. Study Design

The study was a descriptive cross-sectional survey involving field measurements of environmental noise levels at specific recorded geographic coordinates.

### 2.3. Study Population

The overall study population included students above 14 years of age in eight senior secondary schools, especially classes I, II, and III (equivalent to grades 10–12 in USA). A combination of stratified and simple random sampling was employed in getting the appropriate sample population for the study described in this paper. Data collection was conducted in four of the eight secondary schools selected in our overall study. The four schools, which are located in metropolitan Ibadan, were Ikolaba Grammar School (Ikolaba or IGS), Oba Akinbiyi High School (Oba Akinbiyi or OAHS), Anglican Commercial Grammar School (Anglican or ACGS), and Bashorun Ojoo High School (Bashorun Ojoo or BOHS). A total of 400 participants were included in the study, 50 from each of the eight study schools.

### 2.4. Materials and Tools

Well structured questionnaires including a technician walk through or observational check list were administered to collect data related to environmental and health and learning related issues, with a focus on noise and its sources located outdoors and indoors. These were based on previous school-based research in the USA including quantitative and qualitative measurements related to noise loudness and/or frequency [15–17] and environmental epidemiology study design [18]. Informed consent was obtained from school administration and participating students and staff before the study commenced.

The questionnaires consisted of both open and closed ended questions with five sections. The sections were section A for general information about the schools; section B for sociodemographic data on participants, section C for occupational and learning related features of the schools and classrooms, section D for environmental characteristics; and, section E for assessing health and learning related conditions (symptoms). Questionnaires were self administered except for section D, which was an observational checklist used by field technicians to assess the environmental health indicators inside and outside the school environments.

The noise levels were measured using a factory-calibrated TECPEL Model 330 series sound level meter (SLM) set at the slow response mode with A-weighting (A-weighted decibels (dBA)). The measurements were conducted twice, between 9:00-10:00 AM in the morning and 1:00-2:00 PM in the afternoon. Measurements were conducted at two points in the classroom, including where students were seated, and on the play ground. We recorded these data daily for a period of five school days, that is, one full week.

A hand-held, battery-powered factory calibrated global positioning system (GPS) was used to determine the geographic coordinates of the school locations and this study's noise measurements. Traffic density, or the manual count of the number of vehicles (automobiles, vans, and smaller and larger trucks and buses), around the school with the highest noise levels was also determined during the study period. The classroom dimensions—floor space and the sizes of potentially open doors and windows—were also determined (data not reported here) because outdoor sources of air and noise pollution are well known to impact indoor environments in urban and rural areas worldwide.

### 2.5. Statistical Analysis

Data from completed questionnaires were entered in Microsoft Excel spreadsheets and then were imported into the SPSS statistical software package for analyses. Frequency distribution tables and other descriptive statistics such as numbers and percentages were used to summarize study data in both tabular and graphical formats.

## 3. Results

### 3.1. General Information about the Schools

The general information—including some geographical and physical characteristics—obtained about the study schools and classroom buildings isa presented in Table 1. IGS recorded the highest student and overall populations. From the GPS readings the school situated at the highest elevation was IGS, and the four schools were located within about 20 meters (from the main/front entrance) of nearby primary roads. In addition, results from the observational checklist conducted during school hours revealed that the buildings of most of these study schools were old and dilapidated at the time of study. Wall and floor cracks were visible. Most classes lacked finished ceilings, which increased student and staff (teaching and non-teaching) exposure to heat (temperature) and humidity, solar radiation, noise, and outdoor air pollutants. Furthermore, the classrooms appeared to be overcrowded (50–60 students per class) based on a Nigerian policy guideline—occupancy of ≤36 in six rows of six students in a floor area ≥19.4 m^2^ with ≥2 m between the teacher and front row [19]—and had only one door. Classrooms were observed at the times of the study to be usually rowdy, noisy (due to occupants talking), and not conducive to learning. The presence of vehicular traffic around the school environment, however, still likely constituted the major source of noise.

### 3.2. Reported Sources of Environmental Noise at Schools

Our data presented in Table 2 describe how participants at study schools reported noise from vehicular traffic on nearby primary roads as the major sources of noise pollution, except ACGS, where participants reported the major source of noise to be nearby religious houses. Most of the respondents reported that they were affected by noise, especially the students from OAHS and BOHS (98% and 88%, resp.). The proportions of participants who reported that they were affected by noise at other study schools were 76% and 64% at IGS and at ACGS, respectively.

### 3.3. Traffic Density around Schools

**Figure 1:**
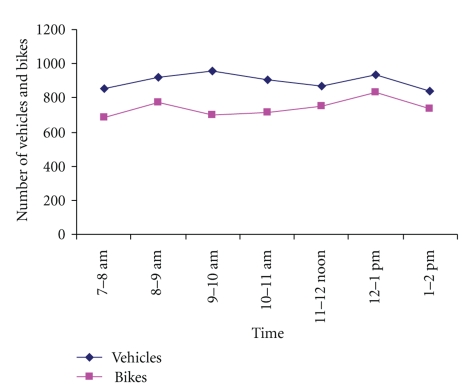
Mean observed five-day traffic density on main roads near OAHS in Ibadan, Nigeria, by hour during school hours. Please note “bikes” in this setting refer to motor bikes only (not bicycles).

Figure 1 presents mean observed five-day traffic density on main roads near one study school, OAHS, by hour during school hours. OAHS was chosen based on the questionnaire results concerning identified sources (please refer to Table 2). Our cross-sectional data suggested that the numbers of motor cars were more than of motor bikes; these types of mobile sources of air pollution and noise far outnumbered smaller and larger trucks during the study period. The five-day mean number of vehicles ranged between 840 and 957 for motor cars and 702 and 832 for motor bikes. The highest frequency (counts) during school hours was observed between 9-10 AM and 12.00-1.00 PM.

### 3.4. Measured Environmental Noise at Schools

**Figure 2:**
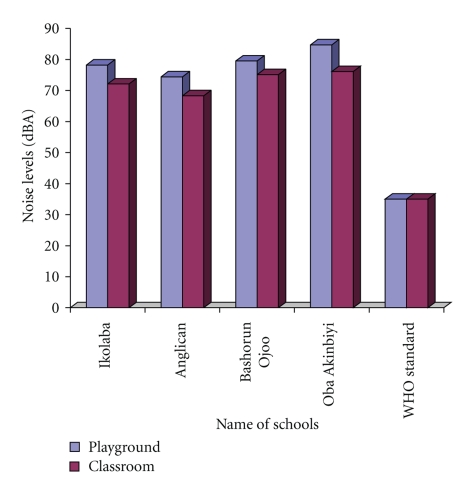
Average school-day hour noise levels indoors (study classrooms) and outdoors (on playground) at selected secondary schools in Ibadan, Nigeria, compared to each other and to the current WHO standard (35 dBA) for community learning environments.

Figure 2 displays the noise levels recorded at different coordinates in the four schools studied. The mean noise levels recorded across the schools were between 68.3 dbA and 84.7 dBA outdoors in the play grounds and between 69.5 dBA and 76.1 dBA inside the classrooms. These ranges of values were beyond the WHO recommended 35 dBA noise level for community learning (school) environments. One of the study classrooms at OAHS (OAHS II) recorded the highest mean noise level, 76.1 dBA for indoor classroom noise; the mean of measurements at this school was 84.7 dBA for outdoor play ground noise. On the other hand, the lowest mean noise level recorded was at ACGS (ACGS II), 68.3 dBA for indoor classroom noise; the mean of measurements at this school was 74.4 dBA for outdoor play ground noise. Noise levels measured were due to reported and identified outdoor sources as well as the people (students and staff) themselves when they were learning indoors or playing outdoors.

### 3.5. Reported Health and Learning-Related Outcomes (Symptoms) due to Noise

**Figure 3:**
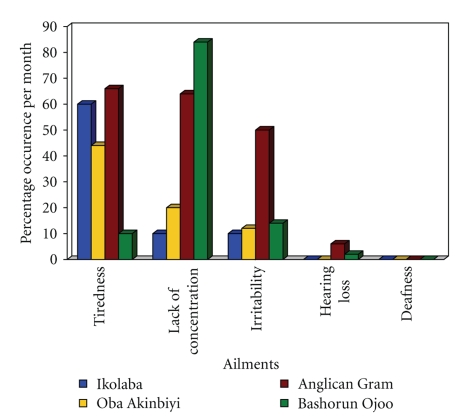
Self-reported noise-related adverse symptoms due to chronic (continuous or intermittent) and acute exposure to noise by students in selected secondary schools located near major roadways, Ibadan, Nigeria. *N* = 200 or *n* = 50 per study school. Please note that “hearing loss” is equivalent to hearing impairment and “deafness” is equal to complete, irreversible hearing loss.

Figure 3 presents self-reported monthly noise-related adverse symptoms by 200 participant students at four study schools. About 60% of the respondents from IGS reported to suffer from tiredness associated with noise. In addition, at IGS, about 10% reported to suffer from a lack of concentration and irritability, but no respondents reported hearing impairment and thus also did not report hearing loss (deafness). At OAHS, about 44% of the respondents reported to suffer from tiredness, 20% from a lack of concentration, and 12% from irritation. No respondents reported hearing impairment and thus also did not report hearing loss. ACGS reported 66%, 64%, 50%, 6%, and 0% for the same categories of health and learning related outcomes, respectively. BOHS reported the most (84%) lack of concentration, but the fewest (2%) reported auditory problems (hearing impairment). Overall, tiredness was found to be the most prevalent noise-associated outcome reported across schools. No participating student or staff member reported being deaf during our study, but our concerns of chronic noise exposure still existed.

## 4. Discussion

The impact of noise on children's health and development in schools is of major public health concern. This could be greatly reduced if noise problems were taken into consideration as early as possible when a school is being designed. Environmentally sustainable buildings designed to enhance resource conservation and indoor air and environmental quality, including minimizing noise, assist education goals.

Each of the four study schools was sited close to main roads, which potentially exposed students to excess and high levels of noise from vehicular traffic, from intermittent use of vehicle horns, from the tires during the sudden use of brakes, and so forth, relative to their individual proximities to the road both inside and outside of buildings (classrooms). We note that noise control devices like absorbers (materials have noise reduction coefficients as well), reflectors, and attenuators were absent in schools studied.

From a comparative perspective, BOHS reported a lack of concentration as the most prevalent health and learning related noise problem (84%). IGS, OAHS, and ACGS, however, reported tiredness as their most prevalent health and learning related noise problem at 60%, 44%, and 66%, respectively. The results obtained and their variations may be traced to the fact that most of the schools are located close to main roads and are readily affected by the noise from vehicle engines in degrees dependant on their individual proximities to the road—mean levels measured during this study indoors and outdoors were between 68.3 dBA and 84.7 dBA.

The cross-sectional mean noise levels measured in this study, though limited with respect to longer-term exposure dose for surveyed students, are proper representations, that is, estimates, of acute (if large or episodic) and/or chronic (continuous or intermittent) exposures experienced by students attending and adults working at secondary schools in this setting. These estimated exposures are capable of initiating and aggravating noise-induced hearing impairment as well as nonauditory health effects specified by WHO (2000) we selected to include in our pilot study. This pilot study's findings also appeared to be consistent with recently completed European Commission funded studies, which investigated road traffic and aircraft noise exposure and potential impacts on child cognition and health as well as the role of a child's increased sensitivity [20–22]. Exposure to noise levels over 55 dBA was demonstrated in those studies in industrialized countries to have interfered with a child's learning process—study children had problems filtering out background noise and interpreting speech and had lower scores in reading tests. Therefore, generally speaking, the noise generated by mobile sources on nearby primary roads could be partially or completely responsible for the various environmental and health and learning related measurements obtained in our pilot study's secondary schools.

Furthermore, our observed absence of noise control measures and devices in the four study schools could also be responsible for the high reported prevalence of health and learning related outcomes. The impact of noise on children's health and development in schools imposes a potentially considerable financial burden, which could be greatly reduced if noise concerns were taken into consideration as early as possible when a school is being planned as well as during operations and maintenance of the buildings and facilities.

This study measured cross-sectional, shorter-term dBA, or loudness, to assess potential exposure at schools to noise. However, though dBA correlates well with human judgment of relative loudness, the metric does not correlate as well with human judgment of relative noisiness or subjective sound quality, that is, comparing sounds with distinct spectral or tonal characteristics including frequency [3]. High-frequency sounds may be relatively more hazardous to human hearing, and high-frequency, intermittent, and impulsive sounds may be more annoying due to their temporal unpredictability. This study did not have the resources to purchase and use sound level meters capable of measurements of both loudness and frequency, but future research can.

## 5. Conclusion

As Nigeria strives towards achieving adequate health care for the populace, the school and learning environment must not be neglected. This study suggested that noise levels indoors (classrooms) and outdoors (playgrounds) across schools were higher than WHO permissible levels for community learning environments. The most reported health problems potentially associated with acute (large or episodic) and/or chronic (continuous or intermittent) exposure to noise within the school environment were lack of concentration and tiredness. Evidence has suggested that noise in learning environments has considerable effects on the learning abilities and the general productivity of children in terms of their academic performance as compared to children in serene learning environments. Therefore, this study should inform future, more rigorous longitudinal research with repeated measures across seasons, indoors and outdoors, in Nigerian schools as well as collaborative efforts by government agencies and education stakeholders for policy formulation and implementation. The goal is to promote enhanced learning environments for children free from excess environmental noise, which will also assist the productivity and improve the health of adult staff.

## Figures and Tables

**Table 1:** General information about four selected secondary schools studied in Ibadan, Nigeria.

School name and acronym	Location of school within Ibadan	Number of students	Number of teaching staff	Number of Non-teaching staff	Geographic coordinates, elevation above sea level (in m)	Distance to road from the main entrance area (in m)
Ikolaba Grammar School, IGS	Agodi Gate	1371	43	14	N07°24.051′ E003°55.285′ 260 m	10
Oba Akinbiyi High School, OAHS	Oremeji	300	34	11	N07°24.712′ E003°53.602′ 226 m	10
Anglican Commercial Grammar School, ACGS	Yemetu	600	28	10	N07°23.750′ E003°54.126′ 215 m	20
Bashorun Ojoo High School, BOHS	Ashi Road	612	42	10	N07°25.341′ E003°56.066′ 253 m	10

**Table 2:** Identified sources of environmental noise at four selected secondary schools in Ibadan, Nigeria.

Name of school	Identified sources of noise in the school environment,					
	number of respondents (percentage) by school					
	Vehicles	Market	Factories	Religious houses	Others	Total by school
Ikolaba Grammar School, IGS	47 (94%)	0 (0%)	0 (0%)	0 (0%)	3 (6%)	**50 (100%)**
Oba Akinbiyi High School, OAHS	39 (78%)	3 (6%)	3 (6%)	5 (10%)	0 (0%)	**50 (100%)**
Anglican Commercial Grammar School, ACGS	9 (18%)	6 (12%)	0 (0%)	31 (62%)	4 (8%)	**50 (100%)**
Bashorun Ojoo High School, BOHS	33 (66%)	16 (32%)	0 (0%)	0 (0%)	1 (2%)	**50 (100%)**
Total Across Schools	**128 (64%)**	**25(12.5%)**	**3 (1.5%)**	**36 (18%)**	**8 (4%)**	**200 (100%)**
